# A Novel Triple Reassortment H3N8 Avian Influenza Virus: Characteristics, Pathogenicity, and Transmissibility

**DOI:** 10.1155/2023/6453969

**Published:** 2023-06-30

**Authors:** Sheng Chen, Shuqun Shen, Yutao Teng, Ruoying Li, Xinheng Zhang, Jiajia Liu, Zhiqiang Wu, Zhuanqiang Yan, Feng Chen, Qingmei Xie

**Affiliations:** ^1^Heyuan Branch, Guangdong Provincial Laboratory of Lingnan Modern Agricultural Science and Technology, College of Animal Science, South China Agricultural University, Guangzhou 510642, China; ^2^Guangdong Engineering Research Center for Vector Vaccine of Animal Virus, Guangzhou 510642, China; ^3^South China Collaborative Innovation Center for Poultry Disease Control and Product Safety, Guangzhou 510642, China; ^4^Key Laboratory of Animal Health Aquaculture and Environmental Control, Guangzhou 510642, Guangdong, China; ^5^College of Animal Science Provincial Key Lab of AgroAnimal Genomics and Molecular Breeding, South China Agricultural University, Guangzhou 510642, Guangdong, China; ^6^Southern Medical University Institute for Global Health, Southern Medical University, Guangzhou 510642, China

## Abstract

An increasing number of new subtypes of avian influenza viruses (AIVs) are reported to be infecting humans, including H3N2, H5N1, H7N9, H10N8, and the recently emerged H3N8 virus in China in 2022. However, the genetic and biological properties of the currently prevalent H3N8 AIVs are not yet fully understood. This study reports the isolation of a novel triple reassortment H3N8 virus (GD-H3N8) from chicken flocks in Guangdong province, China, in 2022. The GD-H3N8 virus contains the Eurasian avian duck-origin H3 gene, the North American avian N8 gene, and dynamic internal genes of the H9N2 virus, and shows high homology with human H3N8 strains. The GD-H3N8 isolate has multiple mammalian adaptive mutations associated with receptor binding and virulence. Growth kinetics assays demonstrate that the GD-H3N8 isolate is capable of efficient replication in avian, mammalian, and human cells in vitro. In vivo, the GD-H3N8 isolate can replicate efficiently in mice without preadaptation, in addition to establishing systemic infection and transmission by direct contact in chickens. These findings underscore the need for continued surveillance of H3N8 viruses to identify circulating strains that may potentially threaten human health.

## 1. Introduction

The first case of human infection with a novel avian influenza A virus (AIV) H3N8 was reported on April 26, 2022 in Henan Province, China [1], and a second case was identified soon afterward in Hunan Province, China [2]. Like many other subtypes of influenza A viruses (IAV), wild waterfowl are considered the natural reservoir of H3N8 influenza viruses and are associated with mild to no disease in infected birds [3]. However, H3N8 viruses have a wide host range and have been associated with ongoing outbreaks in equines and dogs [4]. Additionally, H3N8 viruses have been isolated from pigs [5], donkeys [6], ferrets [3], and seals [7], indicating that H3N8 viruses are capable of crossing the species barrier and establishing lineages in mammals. Of particular concern, H3N8 viruses can occasionally cause severe respiratory disease in mammalian hosts and even lead to death. For example, seals along the coast of New England recently died of respiratory pneumonia, and the causative agent was identified as an avian-origin H3N8 influenza virus [7]. Although AIVs are generally considered host-specific, and only a limited number of cases have been documented where they have breached the host barrier to infect humans [8]. But unfortunately, the first case of human infection with an avian-origin H3N8 virus was confirmed in Henan Province, China [1].

The prevalence of H3N8 viruses has been increasing in recent years. Since its first identification in equines in Florida in 1963 [9], the virus has spread across several continents, including Eurasia [10], South America [11], and Australia [12]. H3N8 viruses have been classified into two geographical lineages: the American lineage and the Eurasian lineage [13]. In 1999, H3N8 was first reported to have been transmitted from horses to dogs in the United States [14] and has been circulating since then [15]. Currently, H3N8 is the only IAV subtype circulating in equine and dog species [13]. Notably, equine H3N8 strains have also been detected in swine [5] and donkeys [6] in China. Recently, researchers have found that a wild bird-origin H3N8 virus can be transmitted among mammals through respiratory droplets in ferrets [16]. Furthermore, a recent report provided serological evidence of equine IAV infections among humans who had contact with horses [17]. The increasing frequency of virus identifications and infections caused by H3N8 viruses suggests that caution must be taken regarding this AIV subtype, particularly given the confirmed cases of H3N8 infection in humans. If H3N8 acquires the ability to efficiently transmit from person to person, it could lead to an outbreak of the H3N8 influenza epidemic and potentially even a pandemic among susceptible populations. Therefore, a thorough assessment and monitoring of the prevailing H3N8 strain in poultry are imperative to comprehend the progression of H3N8 viruses that may have pandemic potential.

The present study aimed to conduct a comprehensive assessment of a newly identified avian-origin H3N8 virus with triple reassortment, including its genetic characteristics, transmissibility, pathogenicity, and inflammatory responses. Our genetic analysis revealed that the newly identified H3N8 virus had undergone multiple reassortment events, especially intercontinental reassortment, originating from North America and Eurasia. This virus displayed significant genetic similarity to human H3N8 strains. Further analysis of the amino acid sequence revealed that the newly formed H3N8 virus had acquired several genetic markers linked to receptor binding, pathogenicity in mammals, and virulence. The kinetics assays of multicycle growth showed that the recently emerged H3N8 reassortment virus exhibited efficient replication in vitro in cells of avian, mammalian, and human origin. Although the H3N8 reassortment virus showed mild clinical symptoms in vivo, it demonstrated effective virus transmission and systemic infection in chickens. In experiments conducted on mice, the H3N8 virus undergoing reassortment replicated effectively in both the nasal turbinates and lungs without requiring preadaptation. Our study highlighted that the newly emerged triple reassortant avian-origin H3N8 IAV possesses the potential to pose a significant threat to mammals. This highlights the need for enhanced surveillance of circulating H3N8 IAV in poultry.

## 2. Materials and Methods

### 2.1. Ethics Statem*ent*

All animal experiments were carried out in strict accordance with the Animal Ethics Procedures and Guidelines of the People's Republic of China and the Experimental Animal Welfare Ethics Committee of South China Agricultural University (SYXK-2022-0136).

### 2.2. Viruses and Cells

In January 2022, a minor outbreak of AIV was reported in meat chicken flocks in Guangdong Province. Chickens that have been infected display various respiratory symptoms, such as tracheal obstruction and airsacculitis. The chickens that were infected had received both a bivalent inactivated vaccine for avian influenza (containing H5 and H7 subtypes) and an inactivated vaccine for avian influenza subtype H9. An H3N8 IAV strain was isolated from chickens that were infected. The virus was purified through three rounds limiting dilution in 9-day-old specific pathogen-free (SPF) embryonated chicken eggs (Guangdong Wens Dahuanong Biotechnology Co., Ltd.). The virus was cultured in SPF chicken eggs at 37°C for 48 hr and stored at −80°C for future utilization. The Medin–Darby canine kidney cells (MDCK), human A549 cells, and chicken embryo fibroblast cells (CEF) were maintained in our laboratory and cultured in Dulbecco's modified Eagle's medium (DMEM) (Gibco, USA) supplemented with 10% fetal bovine serum (FBS) (Gibco, USA) at 37°C with 5% CO_2_.

### 2.3. Sequencing and Phylogenetic Analysis

Viral RNA was extracted from infected allantoic fluid using the AxyPrep Body Fluid Viral DNA/RNA Miniprep Kit (Axygen, USA), reverse transcription and cDNA synthesis were performed according to the manufacture's instruction of HiScript II 1st Strand cDNA Synthesis Kit (Vazyme, Nanjing, China). The full-length gene segments were amplified by polymerase chain reaction (PCR) via segment-specific primers and the corresponding primers are shown in *Supplementary 1*. The PCR products cloned into the pMD-19T vector (Takara, Beijing, China) and the positive recombinant were sent to Sangon Biotech (Shanghai) Co., Ltd. to sequencing. The sequencing data were spliced using the SeqMan software of Lasergene package (DNASTAR) and submitted to GenBank (NCBI accession number: ON627696-ON627703). The sequences were compared to those of classical reference influenza viruses based on previous reports. The reference sequences were obtained from the NCBI database (https://www.ncbi.nlm.nih.gov/) and GISAID database (https://gisaid.org/) (*Supplementary 2*). The phylogenetic trees for the eight genes of H3N8 virus were constructed using the maximum likelihood method under the Tamura–Nei model with 1,000 bootstrap replicates in the MEGA-X software.

### 2.4. Growth Kinetics Analysis

MDCK, CEF, and human A549 cells were infected with GD-H3N8 virus at a multiplicity of infection (MOI) of 0.01 and then cultured in maintenance medium at 37°C with 5% CO_2_. The supernatant was collected at 12, 24, 36, 48, 60, and 72 hr postinfection, and cells were determined by calculating the TCID50 using the Reed–Muench method.

### 2.5. Histopathological Analysis

The chicken lung (throat, trachea, kidney, and liver) tissues were collected at Day 3 postinfection (dpi) and subsequently fixed in 10% formalin at room temperature for 120 hr to make paraffin sections with 5 *μ*M in thick. After the process of deparaffinization and hydration, the slides were subjected to staining using hematoxylin and eosin (H&E).

### 2.6. Quantitative Reverse Transcription PCR

The expression levels of cytokine genes were detected using quantitative reverse transcription polymerase chain reaction (qRT–PCR). The RNA from the samples was extracted by using a DNA/RNA extraction kit (Vazyme, Nanjing, China), qPCR experiments were conducted using Taq Pro Universal SYBR qPCR Master Mix (Vazyme, Nanjing, China) in accordance with the manufacturer's guidelines. The amplification primer sequences utilized were documented in *Supplementary 3*.

### 2.7. Chicken Experiments

Twelve, 1-week-old SPF chickens (Guangdong Wens Dahuanong Biotechnology Co., Ltd., China) were intranasally inoculated with 10^6.0^ EID_50_ of H3N8 virus in a 100 *μ*L volume. At 12 hr postinfection, three unexposed chickens were cohoused together with the inoculated chickens, and morbidity and mortality were monitored daily for 14 days. Oropharyngeal and cloacal swabs were collected on Days 3, 5, 7, and 9 postinfection (dpi) for virus titration in 9-day-old chicken embryos. Three chickens of the inoculated group on days 3, 5, and 7 were euthanized, and their heart, liver, spleen, lung, kidney, bursal, trachea, throat, ileum, and contents of ileum were collected for virus titration and pathologic analysis.

### 2.8. Mice Experiments

Eleven, 6-week-old female BALB/c mice (Guangdong Medical Laboratory Animal Center, China) were light anesthetized using CO_2_ and then intranasally inoculated with 10^6.0^ EID_50_ of H3N8 virus in a 50 *μ*L volume. Members of the mock control group were inoculated with 50 *μ*L PBS. Three mice were euthanized on Days 3 and 6 after infection and their nasal turbinate, lung, kidney, spleen, liver, and brain were collected for virus titration. The weight loss and survival of the remaining five mice were monitored daily for 14 days.

### 2.9. Statistical Analysis

All statistical analyses were performed using GraphPad Prism software version 8.0. Statistically significant differences between experiment groups were carried out using the analysis of Student's *t*-test. Differences were considered statistically significant at *p* < 0.05.

## 3. Results

### 3.1. Genetic Characterization of Avian Origin GD-H3N8 Virus

**Figure 1:**
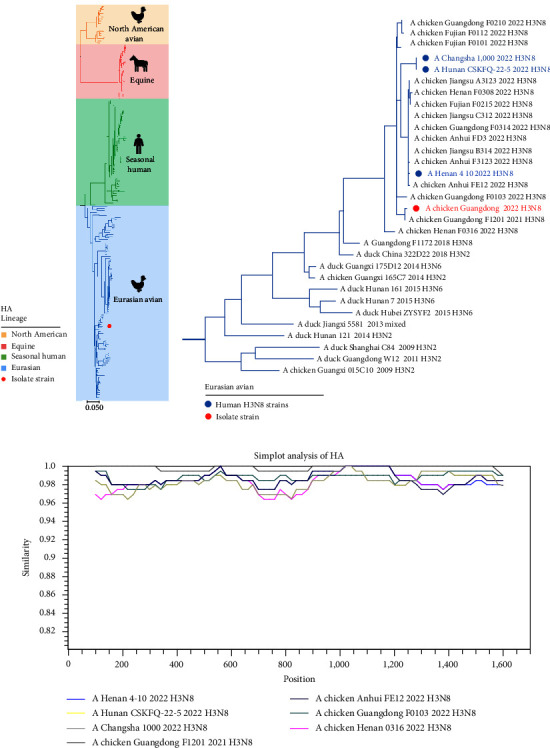
Phylogenetic analysis of hemagglutinin (HA) gene of H3 subtype influenza viruses. (a) Phylogenetic tree of *HA* gene of H3 subtypes. The North American avian lineage is indicated in yellow, the equine lineage in pink, the seasonal human lineage in green, and the Eurasian avian lineage in blue. The laboratory isolate strain is shown in a red solid circle. The scale bar represents the number of nucleotide substitutions per site (subs/site). (b) Phylogenetic tree of the *HA* gene of the Eurasian avian H3 subtype. Human H3N8 viruses are shown in blue solid circles and the isolate strain is shown in a red solid circle. The phylogenetic trees were constructed using the maximum-likelihood method based on the Tamura–Nei model with 1,000 bootstrap replicates in MEGAX. All sequences were downloaded from the Global Initiative on Sharing Avian Influenza Data and the Influenza Virus Resource at the National Center for Biotechnology Information. (c) Similarity plotting analysis of the *HA* gene of the isolate strain. Bootscan evidence of recombination was generated by the Simplot program based on the pairwise distance model with a window size of 200, a step size of 20, and 1,000 bootstrap replicates. The *x*-axis represents the nucleotide position and the *y*-axis represents the similarity of sequences.

In January 2022, a novel IAV H3N8, designated as GD-H3N8, was isolated from infected meat chicken flocks in Guangdong Province, China. These chickens displayed respiratory symptoms such as tracheal obstruction and air sacculitis. Due to recent reports of human infection with H3N8 AIV in China, we conducted a genetic characterization analysis of the avian-origin GD-H3N8 isolate. The overall genetic structure of the global H3 subtype HA gene phylogenetic tree represented four major genetic lineages, including two major geographically dependent avian lineages (Eurasian and North American), equine lineages, and seasonal human lineages (Figure 1(a)). The GD-H3N8 isolate, along with three human isolates, clustered into the Eurasian lineage and were markedly distinct from the other three lineages. Furthermore, these isolates formed an independent phylogenetic branch with a high bootstrap value (100%) with other H3N8 strains recently isolated in China. Our phylogenetic analysis suggested that the independent branch was most closely related to duck-origin H3Ny isolates in southern China (Figure 1(b)). Similarity Plotting demonstrated that the GD-H3N8 isolate exhibited high similarity with the human H3N8 isolates (sequence identity > 96%) (Figure 1(c)). These results imply that the novel GD-H3N8 HA gene has a closer relationship with the duck-origin H3 subtype influenza virus isolated in southern China and has high homology with human H3N8 isolates.

**Figure 2:**
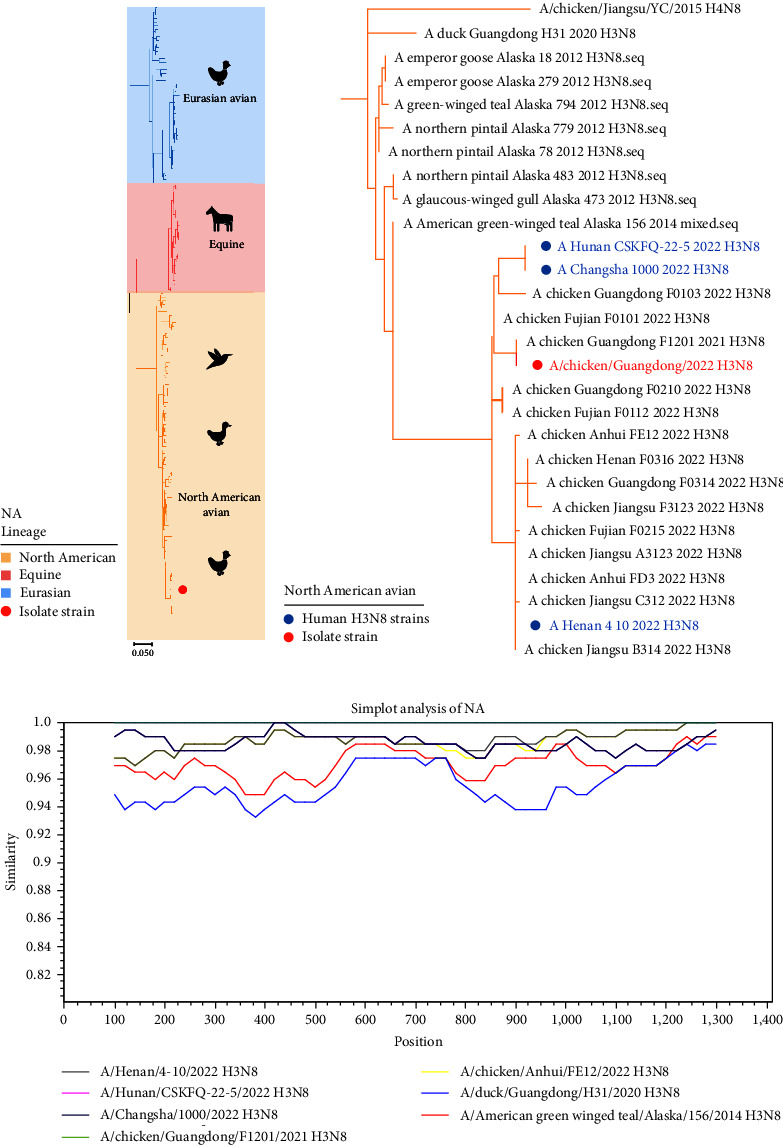
Phylogenetic analysis of neuraminidase (NA) gene of N8 subtype influenza viruses. (a) Phylogenetic tree of *NA* gene of N8 subtypes. Yellow indicates North American avian lineage, pink indicates equine lineage, and blue indicates Eurasian avian lineage. Laboratory isolate strain is shown in red solid circle. The scale bar represents the number of nucleotide substitutions per site (subs/site). (b) Phylogenetic tree of *NA* gene of the North American avian N8 subtype. Human H3N8 viruses are shown in blue solid circle and isolate strain is shown in red solid circle. The phylogenetic trees were constructed by MEGAX using the maximum-likelihood method based on the Tamura–Nei model with 1,000 bootstrap replicates. All the sequences were downloaded from the Global Initiative on Sharing Avian Influenza Data and the Influenza Virus Resource at the National Center for Biotechnology Information. (c) Similarity plotting analysis of *NA* gene of the isolate strain. Bootscan evidence of recombination was based on the pairwise distance model with a window size of 200, step size of 20, and 1,000 bootstrap replicates generated by the Simplot program. The *x*-axis represents the nucleotide position, and the *y*-axis represents similarity of sequences.

What is particularly noteworthy is that the NA gene of the GD-H3N8 isolate belongs to the North American avian lineage, rather than the Eurasian avian lineage, as shown in Figure 2(a). Consistent with the H3 phylogeny, the GD-H3N8 isolate and the three human strains also formed an independent phylogenetic subclade with a bootstrap support of 100% (Figure 2(b)). The N8 gene of these viruses shared a common ancestor with H3N8 waterfowl isolated in Alaska in 2012 and showed the highest similarity to A/green winged teal/Alaska/156/2014 (sequence identity > 94%) based on similarity plotting (Figure 2(c)). These findings provide evidence that the NA gene of the GD-H3N8 isolate and human H3N8 strains originated in Alaska, USA.

All six internal genes of the GD-H3N8 isolate belong to the Eurasian lineage and found to be most closely related to H9N2 virus from birds cocirculating recently in southern and eastern China (*Supplementary 4*). Furthermore, the high homology observed between the six internal genes of the GD-H3N8 isolate and those of human H3N8 strains suggests that this novel virus has the potential to bind to human sialic acid receptors, which are critical for viral entry and replication in human respiratory cells (*Supplementary 5*). This raises concerns about the possibility of the GD-H3N8 virus acquiring the ability to efficiently transmit between humans and cause a potential pandemic. Further studies are needed to assess the pathogenicity and transmissibility of this novel reassortant virus in animal models and to monitor its spread and evolution in the natural reservoir.

**Figure 3:**
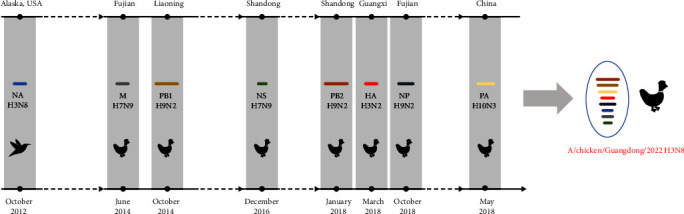
Schematic diagram of the origin of GD-H3N8 virus. The eight gene segments (from top to bottom of the “virion”) are *PB2*, *PB1*, *PA*, *HA*, *NP*, *NA*, *M*, and *NS*. Different colors of each segment represent different virus origins. This diagram is based on nucleotide homology and maximum clade credibility trees.

After conducting a thorough genetic analysis, our findings suggest that the newly discovered GD-H3N8 isolate, which originates from avian species, has undergone multiple reassortment events. Specifically, it appears to have undergone intercontinental reassortment while circulating in the natural reservoir of the virus (Figure 3). Overall, our results indicate that this novel virus is a triple reassortment isolate, containing a Eurasian avian duck-origin *H3* gene, a North American avian *N8* gene, and dynamic internal genes derived from the H9N2 virus. This genetic makeup gives it a high degree of homology with human H3N8 strains, highlighting its potential to cause illness in humans.

### 3.2. Molecular Characteristics of Novel Avian Origin H3N8 Virus

To evaluate the potential risk of the novel triple reassortment H3N8 isolate, we conducted an in-depth analysis of the genetic markers that are known to be associated with mammalian pathogenicity, virulence, or adaptation to new hosts (Table 1). The results showed that the GD-H3N8 isolate exhibited a conserved amino acid sequence at the basic cleavage site (PEKQTR↓GLF), which is important for viral replication and pathogenesis. Notably, the HA protein of the GD-H3N8 isolate did not have the classical substitutions Q226L and G228S, which are commonly found in human H3N8 strains and enhance binding to human-like *α*-2, 6 linked sialic acid receptors [18, 20]. However, it did have A144 and T155 residues in the HA receptor binding pocket, indicating a higher affinity for human-like receptors [22, 23]. In addition, we found no evidence of amino acid deletions in the stalk region of the NA protein, which are known to increase the virulence of AVIs in multiple hosts [24]. The NA protein of the GD-H3N8 isolate had a K435E substitution, which suggests that it would be sensitive to neuraminidase inhibitors but resistant to dextran sulfate. Finally, we did not detect the H274Y substitution in the NA protein, which is associated with resistance to antiviral drugs such as oseltamivir [25, 26].

The polymerase of AIV is a heterotrimer composed of PB1, PB2, and PA, which play a crucial role in determining the host range, tissue tropism, and mammalian pathogenicity of AIV [36]. Notably, mutations associated with mammalian adaptation in PB2, such as G590S, E627K, and D701N, were not observed in the isolate [45, 46]. However, two other mutations, I292V and A588V, were identified in the PB2 protein, which also represent mammalian adaptive markers [37, 38]. Furthermore, a series of key amino acid mutations (I66M, I109V, and I133V, collectively referred to as MVV) were observed in the PB2 protein, which increase the replication efficiency in both avian and mammalian hosts and are seen as the first step for AIV to acquire mammalian pathogenicity [36]. Regarding the PA protein, several adaptive mutations associated with overcoming species barriers and increased virulence were detected, such as L295P, N383D, V476A, A515T, and V630E, while V100A and E382D mutations were not observed [27–30, 47]. Notably, PB1 protein possesses almost all identified mammalian adaptive mutations, including R207K, 269S, I368V, H436Y, L473V, 563R, 622G, and M677T [29, 31–35]. These substitutions are associated with increased polymerase activity, virulence, and decreased antiviral response. Taken together, these findings suggest that the polymerase of the novel triple reassortment GD-H3N8 isolate may have the ability to replicate and cause pathogenicity in mammals.

Several other mutations that enhance viral virulence in mammals were also detected in the GD-H3N8 isolate. These include the N30D, 156D, and T215A mutations in the M1 protein, as well as the P42S mutation in the NS1 protein [39, 40, 43]. Furthermore, the isolate harbored an avian-specific ESEV motif at the C-terminal of its NS1 protein, which has been linked to increased virulence in mice [42]. The presence of an N mutation at position 31 in the M2 protein suggested that the isolate was resistant to the antiviral drugs amantadine and rimantadine [41]. In addition, the GD-H3N8 isolate displayed the D53E mutation in the NP protein, which has been associated with enhanced replication [44]. Overall, our study underscores the importance of continued surveillance and further research to evaluate the potential risk of this novel reassortant virus and to monitor its spread and evolution in the natural reservoir.

### 3.3. Growth Kinetics of Avian-Origin H3N8 Virus

**Figure 4:**
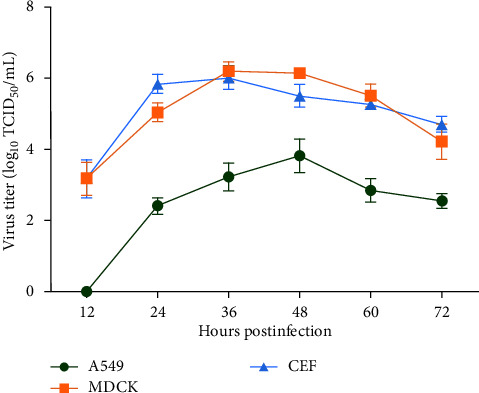
Growth kinetics of GD-H3N8 virus in different cells. MDCK, CEF, and human A549 cells were inoculated with virus at an MOI of 0.01. The supernatants of cells were collected at the indicated time points, and viral titers were titrated in MDCK cells. The experiments were triplicated, and the viral titers at each time point were expressed as mean ± standard deviation.

To assess the replication efficiency of the novel triple reassortment GD-H3N8 isolate in vitro, multicycle growth kinetics assays were conducted in MDCK, CEF, and human A549 cells over a period of 72 hr (Figure 4). The results indicate that the GD-H3N8 isolate replicated efficiently in all three cell types at the indicated time points. Additionally, the GD-H3N8 isolate reached its replication peak earlier in MDCK and CEF cells at 36 hr than in human A549 cells, which peaked at 48 hr. The mean maximum titers of the GD-H3N8 isolate in MDCK and CEF cells were also higher than those in human A549 cells, reaching 6.21 logTCID_50_/mL, 6.04 logTCID_50_/mL, and 3.80 logTCID_50_/mL, respectively. These results suggest that the novel triple reassortment GD-H3N8 isolate has the ability to efficiently replicate in avian, mammalian, and human cells.

### 3.4. Pathogenicity and Transmissibility of Avian-Origin H3N8 Virus in SPF Chicken

Next, we investigated the pathogenicity and transmissibility of the novel triple reassortment GD-H3N8 isolate in SPF chickens. The experimental setup involved intranasal inoculation of the virus into chickens at a dose of 10^6^ EID50, followed by housing of three naive chickens together at 12 hr postinfection. No clinical signs of morbidity or mortality were observed in any of the chickens. We then monitored virus shedding in oropharyngeal and cloacal swabs from both the inoculated and contacted groups (Table 2). In inoculated chickens, virus shedding was observed in oropharyngeal and cloacal swabs as early as 3 dpi, with one of three chickens shedding virus until 9 dpi. The peak mean viral titers were 4.3 ± 0.42 log_10_EID_50_/mL at 5 dpi in oropharyngeal swabs and 3.8 ± 0.16 log_10_EID_50_/mL at 3 dpi in cloacal swabs. In direct-contact chickens, the GD-H3N8 virus transmitted very rapidly to two naive chickens, as virus shedding in oropharyngeal swabs from two chickens was detected at 3, 5, and 7 dpi, and from one chicken at 9 dpi. The peak mean viral titers were 3.5 ± 0.17 log_10_EID_50_/mL at 3 dpi. Only one of the three chickens showed virus shedding in cloacal swabs at 5 dpi. Based on the results of virus shedding, we conclude that the GD-H3N8 isolate can transmit via direct contact at a moderate level in chickens.

**Figure 5:**
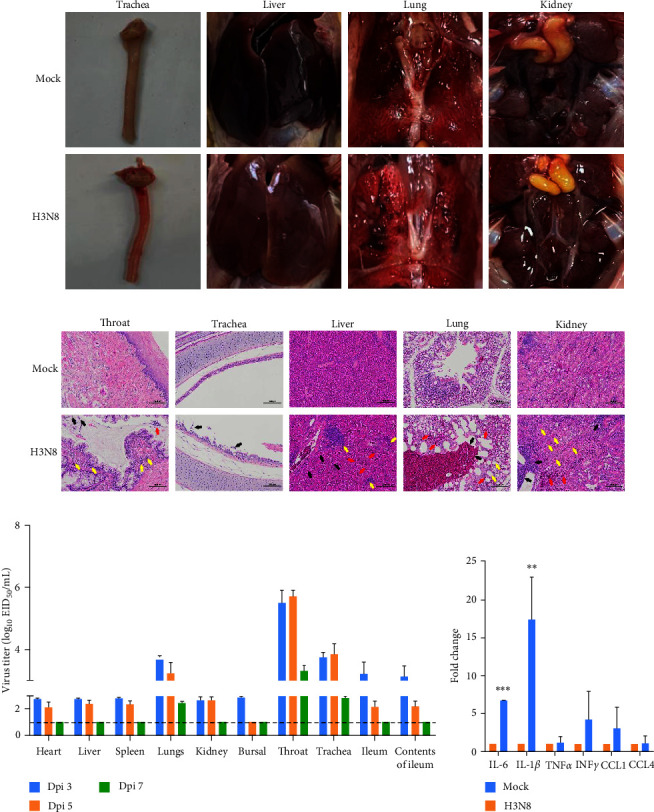
The pathogenicity of GD-H3N8 virus in chickens. (a) Gross changes of chickens intranasally inoculated with GD-H3N8 virus at 3 dpi. Tracheal congestion and mild focal renal lesions. (b) Histopathological changes of hematoxylin and eosin (H&E) stained tissue sections at 3 dpi of GD-H3N8 infected chicken. Magnification, ×200. (c) Tissue distribution of the GD-H3N8 virus in chickens at 3, 5, and 7 dpi. The data shown are the average titers of three chickens. The error bars indicate standard deviations. The dashed black line indicates the lower limit of detection. (d) Proinflammatory cytokines and chemokines in the lungs of infected chickens at 3 dpi. The relative expression levels were determined by RT–PCR. The data shown are the average values of three mice. The error bars indicate standard deviations.  ^*∗∗*^*p* < 0.01,  ^*∗∗∗*^*p* < 0.001.

No significant clinical syndromes in the infected chickens or gross lesions were found in the indicated tissues. Except for mild congestion of upper respiratory tract infection and focal necrosis of the kidney was observed at 3 dpi (Figure 5(a)). At 5 and 7 dpi, the tissue lesions were not obvious (data not shown). To more clearly observe the pathological changes in the tissues of chickens infected by the GD-H3N8 isolate, histopathological examination was performed (Figure 5(b)). In the throat and trachea, infected chickens exhibited upper respiratory tract infection symptoms such as shedding of epithelial and goblet cells, exudation of inflammatory cells in glandular lumens, and infiltration of inflammatory cells around glands. The liver tissues showed extensive sinus and venous congestion along with inflammatory cell infiltration. The kidney biopsy showed renal capillary telangiectasia, renal interstitial inflammation, and focal necrosis. The lungs exhibited local parabronchus and interstitial hemorrhage, respiratory capillary dilatation, and inflammatory cells. Overall, these findings suggest that the GD-H3N8 isolate can cause moderate tissue damage in infected chickens, which could potentially affect their health and productivity.

To investigate the spread of the GD-H3N8 virus in infected chickens, virus titrations in internal organs were conducted (Figure 5(c)). The results indicated that the virus was able to spread systemically in infected chickens at 3 and 5 days postinfection, but was cleared by Day 7. Throughout the observation period, the GD-H3N8 isolate demonstrated effective replication in the lung, throat, and trachea of infected chickens. The peak viral titer in the throat is up to 5.94 log_10_EID_50_/mL at 3 dpi and 5.92 log_10_EID_50_/mL at 5 dpi, which is almost not degraded. A similar situation was observed in the trachea, indicating a consistent infection in the upper respiratory tract. The virus titer in the lung was moderate, but significantly higher than that in other organs. Interestingly, one of the three infected chickens showed a statistical difference in the ileum and ileum content, suggesting a potential injury in the ileum tissue. Overall, these results indicate that although all infected chickens exhibited mild clinical symptoms, the GD-H3N8 virus showed efficient virus dissemination and systemic infection, with viral replication observed in all internal organs.

The different subtype AIVs can induce different levels of innate immune imbalance in poultry or mammals, referred to as a “cytokine storm”, which is a key factor for pathogenicity of AIVs [48, 49]. To understand the effect of the GD-H3N8 isolate-induced cytokine levels, the expression of IL-6, IL1*β*, TNF*α*, IFN-*γ*, CCL1, and CCL4 in the lungs of infected chickens were detected at 3 dpi (Figure 5(d)). We found that IL-6 and IL1*β* in the infection group were much higher than those in the control group by ∼7 fold and ∼17 fold, respectively. However, the increase in inflammatory levels was relatively mild compared to highly pathogenic AIVs, which induced several hundred folds [50].

### 3.5. Pathogenicity of Avian-Origin H3N8 Virus in Mice

**Figure 6:**
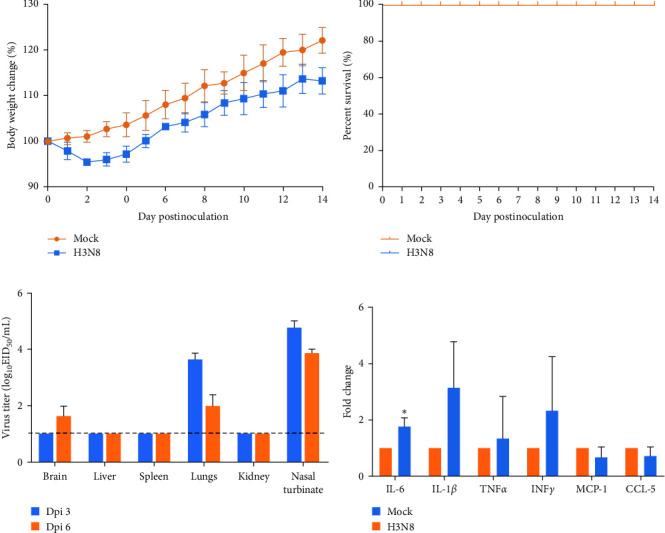
The pathogenicity of GD-H3N8 virus in mice. BALB/c mice were challenged with 10^6^ EID_50_ virus. Body weight (a) and survival (b) were observed for 14 days. (c) Virus titers in indicated tissues were determined in 9-day-old SPF embryonated eggs. The dashed black line indicates the lower limit of detection. (d) Proinflammatory cytokines and chemokines in the lungs of infected mice at 3 dpi. The data shown are the average values of three mice. The error bars indicate standard deviations.  ^*∗*^*p* < 0.05.

To further explore the replication and virulence of the novel triple reassortment GD-H3N8 isolate in mammals, eleven 6-week-old BALB/c mice were intranasally inoculated with 10^6^ EID_50_ of the GD-H3N8 isolate, and morbidity (as measured by weight loss) and mortality were monitored daily for 14 days. No deaths were observed among mice inoculated with the GD-H3N8 isolate, and the majority of mice gradually gained weight during the observation period, with a slight decrease in the early stages (Figures 6(a) and 6(b)). However, the growth rates of the inoculated mice were generally lower than those of the control group, and the final weight was around 10% lower than that of the control group.

To assess the ability of the GD-H3N8 isolate to replicate in mouse organs, we evaluated viral titers in the brain, liver, spleen, lung, kidney, and nasal turbinate at 3 and 6 dpi (Figure 6(c)). The results indicated that the GD-H3N8 isolate replicated efficiently in both the lung and nasal turbinate without preadaptation, with viral titers ranging from 2.42 to 3.89 log_10_EID_50_/mL and 4.00 to 5.02 log_10_EID_50_/mL, respectively. Viral titers were only detectable in the brain of one mouse at 6 dpi (2.02 log_10_EID_50_/mL), and were not detected in the liver, spleen, or kidney of any mice throughout the trial. To gain further insight into the virulence of the GD-H3N8 isolate in mice, we also investigated the inflammatory responses of several representative proinflammatory cytokines and chemokines, including IL-6, IL1*β*, TNF*α*, IFN-*γ*, MCP-1, and CCL-5 in the lung of inoculated mice at 3 dpi (Figure 6(d)). Only IL-6 levels were slightly increased after infection at 3 dpi, suggesting that the GD-H3N8 isolate did not induce a severe inflammatory response in the lungs of mice.

## 4. Discussion

An increasing number of new subtypes of AVIs have been reported to infect humans, including H3N2 viruses since 1970s [51], H5N1 and H9N2 viruses since 1997 [52, 53], and H7N7 viruses in 2003 [54, 55], and the newly emerged H6N1 [56], H7N9 [57], H10N3 [58], H10N8 [59], and H5N6 [60] in China. Recently, the first case of human infection with the novel avian H3N8 virus was reported in Henan Province, China, in 2022 [1], and a second case was identified soon afterward in Hunan Province, China [2], which increases concerns about the potential risk of the H3N8 influenza virus to humans. Previous studies have showed that H3Ny viruses are cocirculating in waterfowl, but no H3N8 virus was detected in chicken flocks in China [61]. However, several reassortment H3N8 viruses were recently isolated from diseased chicken, indicating that the novel H3N8 viruses have adapted to chickens and are pathogenic [2, 62, 63].

In January 2022, a novel H3N8 virus, A/chicken/Guangdong_01/2022(H3N8), was isolated from diseased chicken in Guangdong Province in the present study. The GD-H3N8 isolate shared a high-sequence identity (>96%) in both the H3 and N8 genes with human H3N8 strains, and was a triple reassortment isolate with the Eurasian avian duck-origin H3 gene, North American avian N8 gene, and internal genes of H9N2 viruses. The genetic diversity of this novel H3N8 virus may have arisen from poultry trade or wild bird migration, which are known to play key roles in the maintenance and dissemination of IAVs. For example, recent outbreaks of highly pathogenic H5N8 viruses in Europe were linked to migratory waterfowl [64, 65], and previous reports have shown that wild birds carrying H7 subtype AIV have contributed to the emergence of highly pathogenic viruses [66]. The introduction of the North American avian N8 gene into China was also traced back to wild bird migration in 1992 [67], and has since become the predominant lineage of the N8 subtype [18]. A retrospective investigation found that the novel H3N8 virus causing human infection originated from wild birds and has been endemic in chicken flocks in southern China since December 2021 [2]. These findings suggest that waterbird migration plays a crucial role in the multiple recombination, cross-species transmission, and highly pathogenic variation of AIV, highlighting the need for further studies on the pathogenicity and transmissibility of this new branch strain in vitro and in vivo.

To determine whether the novel GD-H3N8 isolate has enhanced abilities of replication and pathogenicity, we conducted a preliminary evaluation both in vitro and in vivo. Efficient replication in mammalian cells is a crucial factor for cross-species transmission of IAVs. Our results indicated that the GD-H3N8 virus was capable of efficient replication in CEF, MDCK, and human A549 cells. However, significantly lower viral titers of the isolate were observed in human A549 cells, suggesting that the current isolate may not be fully capable of infecting humans.

In vivo experiments demonstrated that the GD-H3N8 isolate could replicate systemically in chickens without obvious clinical signs, but the viruses could be transmitted to chickens by direct contact. Similarly, the GD-H3N8 isolate was able to establish effective infection in mice without preadaptation. Viral replication was observed in all internal organs of infected chickens, while it was only recovered from the nasal turbinate and lungs of mice. Moreover, the virus exhibited enhanced replication in the upper respiratory tract than in the lungs in both chickens and mice. However, the virus showed low pathogenicity and only caused mild clinical symptoms in experimental animals.

In addition to viral transmissibility and replicability, another crucial factor affecting the severity of influenza virus infection is the host immune response. Upon infection with AIVs, most organisms experience a hyperinduction of proinflammatory cytokines and chemokines, resulting in a “cytokine storm” that can cause tissue injury. Previous studies have linked excessive cytokines and chemokines, such as TNF-*α*, IL-6, and IFN-*γ*, to lung damage in AIV-infected individuals, including those infected with H7N9, H5N1, and H5N6 viruses [50]. However, the inflammatory responses induced by H3N8 AIV infection in chickens and mammals remain poorly understood. Our results indicate that moderate levels of inflammatory responses were observed in the lungs of chickens infected with the GD-H3N8 isolate at 3 dpi, with a mild ∼10 fold increase compared to the hundreds of folds induced by highly pathogenic AIVs. This is consistent with the mild clinical symptoms observed in chickens infected with the GD-H3N8 isolate and previous research reports [68]. In the lungs of mice infected with the GD-H3N8 isolate, only IL-6 levels were slightly elevated at 3 dpi, indicating that the virus induced a relatively low level of inflammatory response.

To summarize, our study has demonstrated that a novel triple reassortment avian H3N8 influenza virus isolated in Guangdong province has the potential for zoonotic transmission. This virus showed high homology with human H3N8 strains and had multiple adaptive mutations associated with receptor binding and virulence. Our in vitro experiments revealed that the virus was capable of efficient replication in avian, mammalian, and human cells. In vivo experiments showed that the virus could establish systemic infection and transmission by contact in chickens, and efficiently replicate in mice without preadaptation. Our findings suggest that continued surveillance of H3N8 viruses, especially the newly emerged strains, is essential to identify circulating viruses that may pose a potential threat to human health.

## Figures and Tables

**Table 1:** Key molecular marker of novel reassortment GD/H3N8 virus.

Viral protein	Amino acid position/motif^†^	Phenotypic consequences	References
HA	PEKQTR⬇GLF	Basic cleavage site of low-pathogenicity influenza virus	[18, 19]
	226-228: QSG	Prefer to avian receptor binding	[18, 20, 21]
	134-138: GGSGA		
	T144A, I155T	Increase the binding capacity to human-type *α*2, 6-linked receptors and transmissibility among guinea pigs/ferrets	[22, 23]

NA	No stalk region deletion	Stalk region deletion increase the virulence in multiple hosts	[24]
	H274Y, K435E	Sensitive to neuraminidase but resistant to dextran sulfate	[25, 26]

PA	L295P	Increase virulence in mice	[27]
	N383D	Increases the polymerase activity in both avian and human cells	[28]
	A515T	Increase pathogenic in ducks	[29]
	V476A, V630E	Increase polymerase activity and pathogenicity	[30]

PB1	R207K, H436Y	Increase pathogenic in ducks	[29]
	269S, I368V	Increase virulence	[31]
	L473V	Increase polymerase activity in mammalian cells and in mice	[32]
	563R	Increase virulence and modulation of the host-antiviral IFN response in cells	[33]
	622G	Increase virulence in mice	[34]
	677T	Increase the replication efficiency in mice	[35]

PB2	I66M, I109V, I133V	Increase the replication efficiency in both avian and mammalian hosts	[36]
	I292V	Increase viral polymerase function and attenuates IFN-*β* induction in cells	[37]
	A588V	Increase polymerase activity and replication in mammalian and avian cells; Increase virulence in mice	[38]

M	N30D, T215A	Increase pathogenicity in mice	[39]
	156D	Increase H7N9 virus efficient transmission in guinea pigs	[40]
	S31N (M2)	Decreased inhibition by amantadine	[41]

NS	C-terminal ESEV	Increase viral virulence in mice	[42]
	P42S	Decrease host cell interferon induction	[43]

NP	D53E	Increase replication of viruses with 2009/H1N1-origin NP in swine	[44]

^†^H3 numbering.

**Table 2:** Virus titers at 3, 5, 7 and 9 dpi for inoculated and contacted group.^†^

Group	Swab	Virus shedding on indicated dpi (log_10_EID_50_/mL ± SD)			
		3	5	7	9
Inoculated	Oropharyngeal	3.6 ± 0.05 (3/3)	4.3 ± 0.42 (3/3)	3.5 ± 0.22 (3/3)	3.3 (1/3)
	Cloacal	3.8 ± 0.16 (3/3)	3.7 ± 0.08 (3/3)	3.5 (1/3)	3.4 (1/3)

Direct contact	Oropharyngeal	3.5 ± 0.17 (2/3)	3.2 ± 0.16 (2/3)	3.0 ± 0.10 (2/3)	2.8 (1/3)
	Cloacal	(0/3)	2.8 (1/3)	(0/3)	(0/3)

^†^SPF chicken were intranasally inoculated with 10^6.0^ EID_50_ virus, and oropharyngeal and cloacal swabs were collected on the indicated days. Abbreviations: dpi, day postinfection; EID_50_, 50% egg infective dose.

## Data Availability

The data that support the findings of this study are openly available in NCBI at https://www.ncbi.nlm.nih.gov/, reference number ON627696-ON627703.

## Supplementary Materials

Supplementary 1 H3N8 sequence-specific primers.

Supplementary 2 NCBI or GISAID accession numbers of all strains used in the phylogenetic analysis.

Supplementary 3 Primers for cytokine genes.

Supplementary 4 Phylogenetic analysis of six internal genes of GD-H3N8 virus.

Supplementary 5 Phylogenetic analysis and comparison of internal genes of GD-H3N8 virus and human H3N8 strains.
