# Supplementary material for: A Novel Triple Reassortment H3N8 Avian Influenza Virus: Characteristics, Pathogenicity, and Transmissibility
Source: Transbound Emerg Dis. 2023 Jun 30;2023:6453969. doi: 10.1155/2023/6453969 (PMC12017217; doi:10.1155/2023/6453969)
Supplement: Supplementary 1 — H3N8 sequence-specific primers. [file 6453969.f1.docx]

**Table S1.** H3N8 sequence-specific primers

| Primer | Sequence (5’-3’) |
| --- | --- |
| HA-F | ATGAAAACCGTCATTGCT |
| HA-R | TCAAATGCAAATGTTGCACCT |
| NA-F | ATGAATCCAAATCAAAAAATAATAACC |
| NA-R | TTATCGATGTCAAAGGGAAGAATA |
| PB1-F | ATGGATGTCAATCCGACTTT |
| PB1-R | CTATTTTTGCCGTCTGAGCT |
| PB2-F | ATGGAGAGAATAAAAGAACT |
| PB2-R | CTAATTGATGGCCATCCGAAT |
| PA-F | ATGGAAGACTTTGTGCGACA |
| PA-R | CTATCTCAGTGCATGTGTGAGGAAG |
| M-F | AAAGATGAGCCTTCTTACCGAG |
| M-R | TTACTCCAGCTCTATGTTGA |
| NS-F | ATGGATTCCAACACTGTGTC |
| NS-R | TCAAACTTCTGACTCAGTTG |
| NP-F | ATGGCGTCTCAAGGCACCAA |
| NP-R | TTAACTGTCAAACTCCTCAGC |
