# Supplementary material for: A Novel Triple Reassortment H3N8 Avian Influenza Virus: Characteristics, Pathogenicity, and Transmissibility
Source: Transbound Emerg Dis. 2023 Jun 30;2023:6453969. doi: 10.1155/2023/6453969 (PMC12017217; doi:10.1155/2023/6453969)
Supplement: Supplementary 3 — Primers for cytokine genes. [file 6453969.f3.docx]

**Table S2.** Primers for cytokine genes

| Primer ^a^ | Sequence (5’-3’) |
| --- | --- |
| C-IL-6 | F: AATCCCTCCTCGCCTTTCTG; R: GCCCTCACGGTCTTCTCCAT |
| C-IL-1β | F: ACTGGGCATCAAGGGCTACA; R: GCTGTCCAGGCGGTAGAAGA |
| C-TNF-α | F: GCCCTTCCTGTAACCAGATG; R: ACACGACAGCCAAGTCAACG |
| C-IFN-γ | F: GAGCCAGATTGTTTCGATGTACTTG; R: CATCAGGAAGGTTGTTTTTCAGAG |
| C-CCL1 | F: TACAGCTCCTGCTGCTACAAG; R: CACGATAATAGCTCTGCGGGA |
| C-CCL4 | F: CTCATGCTGGCGTTGTGTTC; R: TGTAGTCCTGTACCCAGTCGT |
| C-GAPDH | F: CCCCCATGTTTGTGATGGGT; R: TGATGGCATGGACAGTGGTC |
| M-IL-6 | F: CCACTTCACAAGTCGGAGGCTTA; R: CCAGTTTGGTAGCATCCATCATTTC |
| M-IL-1β | F: GAGCACCTTCTTTTCCTTCATCTT; R: TCACACACCAGCAGGTTATCATC |
| M-TNF-α | F: ATCCGCGACGTGGAACTG; R: ACCGCCTGGAGTTCTGGAA |
| M-IFN-γ | F: TATCTGGAGGAACTGGC; R: TGCTGGATCTGTGGG |
| M-MCP1 | F: TCTGCCCTAAGGTCTTCAGCA; R: GCATCACAGTCCGAGTCACACTA |
| M-CCL5 | F: CCCTCACCATCATCCTCACT; R: CTTCTTCTCTGGGTTGGCAC |
| M-ACTB | F: GCTCTGGCTCCTAGCACCAT; R: GCCACCGATCCACACAGAGT |

^a^ C: Chicken; M: Mouse
