# Supplementary material for: A Novel Triple Reassortment H3N8 Avian Influenza Virus: Characteristics, Pathogenicity, and Transmissibility
Source: Transbound Emerg Dis. 2023 Jun 30;2023:6453969. doi: 10.1155/2023/6453969 (PMC12017217; doi:10.1155/2023/6453969)
Supplement: Supplementary 4 — Phylogenetic analysis of six internal genes of GD-H3N8 virus. [file 6453969.f4.pdf]

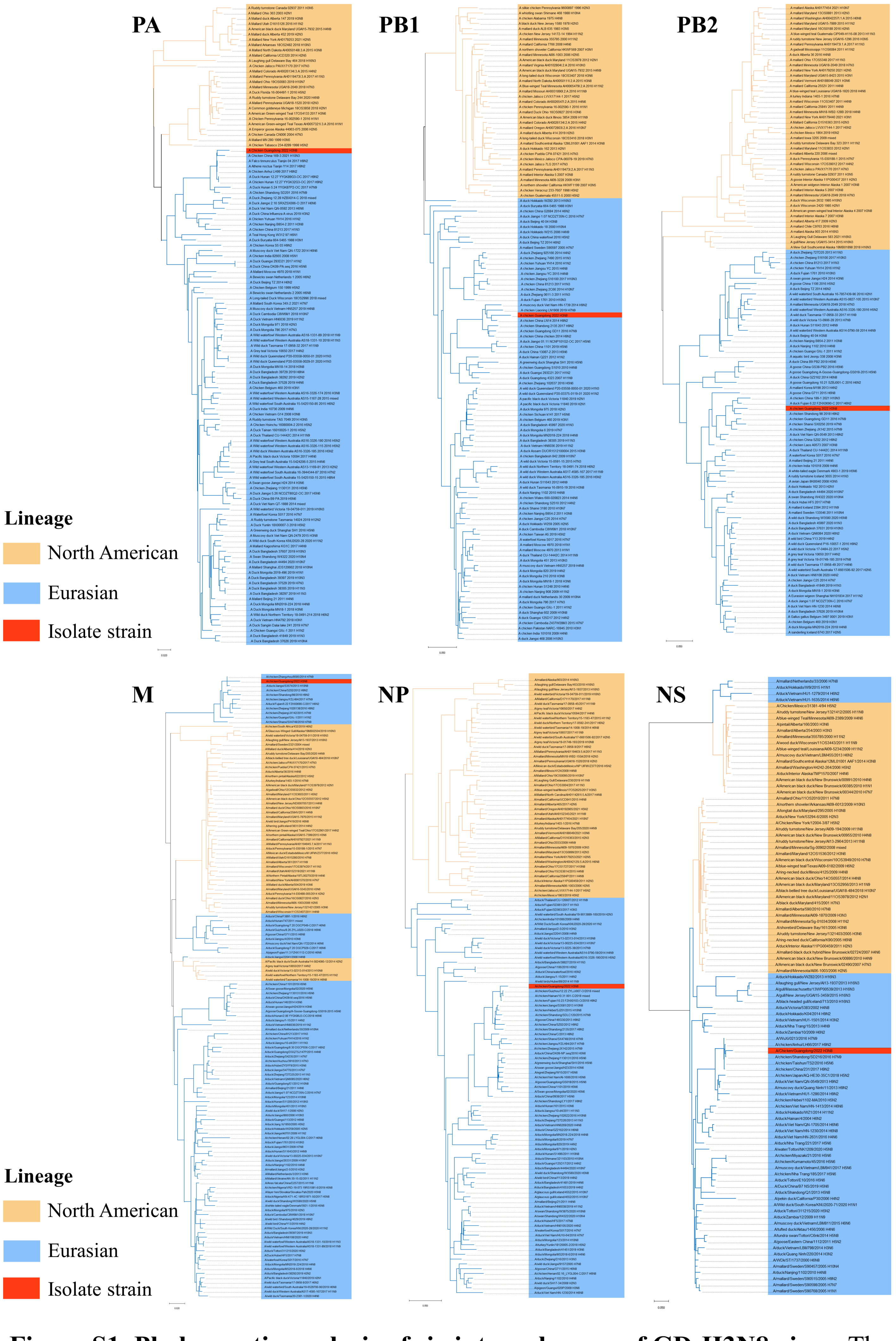

Figure S1. Phylogenetic analysis of six internal genes of GD-H3N8 virus. The phylogenetic trees were constructed by MEGAX using the maximum-likelihood method based on the Tamura-Nei model with 1000 bootstrap replicates. Isolated viruses belonging to different lineages and subtypes are distinguished by colors. The laboratory isolate strain is shown in red.
