# Supplementary material for: A Novel Triple Reassortment H3N8 Avian Influenza Virus: Characteristics, Pathogenicity, and Transmissibility
Source: Transbound Emerg Dis. 2023 Jun 30;2023:6453969. doi: 10.1155/2023/6453969 (PMC12017217; doi:10.1155/2023/6453969)
Supplement: Supplementary 5 — Phylogenetic analysis and comparison of internal genes of GD-H3N8 virus and human H3N8 strains. [file 6453969.f5.pdf]

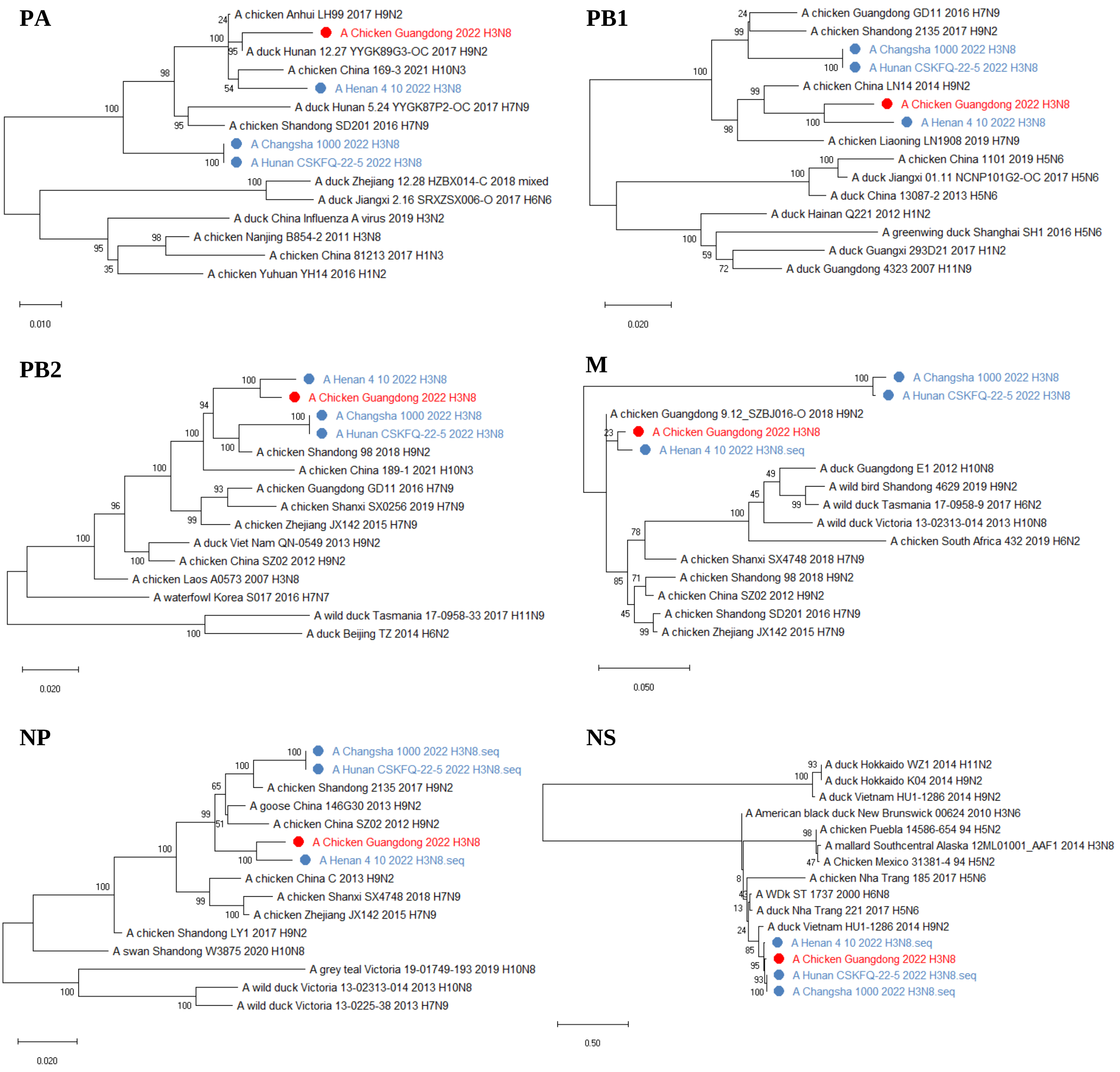

Figure S2. Phylogenetic analysis and comparison of internal genes of GD-H3N8 virus and human H3N8 strains. Phylogenetic tree of *PA*, *PB1*, *PB2*, *M*, *NP*, *NS* genes of the GD-H3N8 virus and human H3N8 strains. Human H3N8 viruses are shown in blue solid circle, and the isolate strain is shown in a red solid circle. The phylogenetic trees were constructed by MEGAX using the maximum-likelihood method based on the Tamura-Nei model with 1000 bootstrap replicates. All the sequences were downloaded from the Global Initiative on Sharing Avian Influenza Data and the Influenza Virus Resource at the National Center for Biotechnology Information.
